# Clinical utility of reticulocyte hemoglobin equivalent in patients with heart failure

**DOI:** 10.1038/s41598-022-18192-x

**Published:** 2022-08-17

**Authors:** Saki Tahara, Yoshiro Naito, Keisuke Okuno, Seiki Yasumura, Tetsuo Horimatsu, Junichi Ohno, Isamu Sunayama, Yuki Matsumoto, Eri Manabe, Kumiko Masai, Kohei Azuma, Koichi Nishimura, Kyung-Duk Min, Akiko Goda, Masanori Asakura, Masaharu Ishihara

**Affiliations:** Department of Cardiovascular and Renal Medicine, School of Medicine, Hyogo Medical University, 1-1 Mukogawa, Nishinomiya, 663-8501 Japan

**Keywords:** Diagnostic markers, Prognostic markers

## Abstract

Anemia and iron deficiency (ID) are common in patients with heart failure (HF) and intravenous (IV) administration of iron to patients hospitalized for decompensated HF with ID improves outcome. The diagnosis of ID in routine practice is based on serum ferritin and transferrin saturation (TSAT) but both have limitations; alternatives should be considered. Reticulocyte hemoglobin equivalent (Ret-He) reflects iron content in reticulocytes but its clinical utility in patients with HF remains uncertain. We prospectively enrolled 142 patients hospitalized for decompensated HF. Sixty five percent had ID as defined in current international guidelines. Ret-He was directly correlated with serum iron and ferritin concentrations and with TSAT. There was a poor relationship between quartile of Ret-He and HF hospitalization or death but increases or decreases in Ret-He between admission and discharge were associated with a worse outcome. The clinical utility of Ret-He for identifying ID and predicting response to IV iron and prognosis for patients with HF requires further investigation.

## Introduction

Anemia is common in patients with heart failure (HF)^1,2^. Iron deficiency (ID) is also frequent, occurring in 35–55% of patients with HF, regardless of the presence or absence of anemia^3,4^. In particular, ID is more common in advanced HF and associated with an adverse prognosis^3,5^. Using the gold-standard for diagnosing ID by bone marrow biopsy, the prevalence rate of iron deficiency anemia (IDA) is reported to be 73% in patients with advanced HF^6^.

In clinical, serum iron, ferritin, total iron binding capacity (TIBC), and transferrin saturation (TSAT) are used for the diagnosis of ID. There are two ID subtypes. One is absolute ID defined by depleted iron stores, and the other is functional ID defined by adequate iron stores but low iron availability for erythroid precursors.

In patients with HF, the most commonly used biomarkers to evaluate iron status are serum ferritin and TSAT. According to current international guidelines on HF, the cut-off values of ID are serum ferritin levels < 100 μg/L or serum ferritin levels 100–299 μg/L with TSAT < 20%^7,8^. However, clinical use of this evaluation is a little complicated, and the best cut-off index for ID in HF patients remains completely unclear.

Reticulocyte hemoglobin equivalent (Ret-He), which can be measured by the automated hematology analyzers, is considered to reflect iron content in reticulocytes^9^. Reticulocytes develop into mature erythrocytes within two days in peripheral blood. Due to the short duration of the reticulocyte stage in erythropoiesis, Ret-He is considered as a direct index of iron availability in erythropoiesis^10^. In addition, usefulness of Ret-He is reported as a screening marker for ID^11^.

However, it has been entirely unknown whether Ret-He can be used as a marker for ID in patients with HF. Therefore, in this study, we investigated the clinical utility of Ret-He in HF patients.

## Results

### Patients characteristics

The patient characteristics on admission are shown in Table 1. Of 142 patients, the median age of patients was 77 years and 58% of patients was men. The median left ventricular ejection fraction (LVEF) and brain natriuretic peptide (BNP) levels were 36% and 659 pg/mL. The median hemoglobin (Hb), serum iron, and TSAT were 10.6 g/dL, 40 µg/dL, and 15.8%. The median Ret-He levels was 32.0 pg on admission. According to quartile of Ret-He levels, the prevalence of hypertension and the use of loop diuretics were different among the groups.

**Table 1:** Patient characteristics on admission according to quartile of Ret-He levels.

Variable	Overall (*n* = 142)	1st quartile (*n* = 36)	2nd quartile (*n* = 37)	3rd quartile (*n* = 33)	4th quartile (*n* = 36)	*P* value
Age (years)	77 (70–83)	75 (69–85)	78 (69–82)	82 (76–86)	76 (73–79)	0.079
Men, n (%)	82 (58)	19 (53)	24 (65)	21 (64)	19 (53)	0.584
BMI (kg/m^2^)	23.2 (20.7–25.1)	23.4 (21.2–25.9)	23.2 (20.7–24.7)	23.8 (21.9–26.2)	21.5 (19.6–24.0)	0.089
SBP (mmHg)	128 (111–143)	130 (120–137)	124 (107–140)	126 (116–142)	129 (105–143)	0.881
HR (bpm)	82 (66–98)	82 (74–96)	82 (67–99)	73 (61–90)	87 (68–106)	0.278
NYHA functional class III/IV, *n* (%)	132 (93)	32 (89)	36 (97)	31 (94)	33 (92)	0.549
Past HF, *n* (%)	66 (46)	18 (50)	19 (51)	9 (27)	15 (42)	0.163
HT, *n* (%)	105 (74)	24 (67)	26 (70)	9 (27)	27 (75)	< 0.001
DM, *n* (%)	62 (44)	14 (39)	18 (49)	9 (27)	10 (28)	0.053
AF, *n* (%)	46 (32)	11 (31)	10 (27)	9 (27)	12 (33)	0.928
OMI, *n* (%)	46 (32)	12 (33)	13 (35)	13 (39)	8 (22)	0.461
CVD, *n* (%)	18 (13)	5 (14)	7 (19)	3 (9)	5 (14)	0.706
eGFR (mL/min/1.73m^2^)	49 (31–68)	56 (35–72)	49 (26–64)	48 (32–60)	48 (24–70)	0.389
LVEF (%)	36 (28–55)	38 (27–57)	36 (30–45)	40 (31–59)	35 (27–54)	0.737
BNP (pg/mL)	659 (428–1238)	724 (480–1185)	744 (458–1510)	532 (423–910)	791 (401–1305)	0.500
Alb (g/dL)	3.3 (3.0–3.6)	3.3 (2.9–3.5)	3.2 (3.0–3.5)	3.5 (3.1–3.7)	3.3 (2.9–3.9)	0.613
Hb (g/dL)	10.6 (9.3–11.9)	9.8 (9.0–10.7)	10.5 (9.7–11.5)	10.8 (10.0–11.7)	11.1 (9.2–13.3)	0.017
MCV (fL)	92.5 (88.2–96.9)	85.6 (79.0–88.7)	91.4 (88.9–94.7)	94.8 (91.3–97.7)	97.1 (94.7–99.9)	< 0.001
MCH (pg)	30.5 (28.6–32.2)	27.0 (24.5–28.8)	30.3 (28.9–31.2)	31.1 (30.1–32.3)	32.3 (31.1–33.9)	< 0.001
MCHC (%)	32.7 (31.9–33.6)	31.5 (30.7–32.3)	32.9 (32.3–33.6)	33.1 (32.5–33.6)	33.1 (32.7–34.1)	< 0.001
Fe (µg/dL)	40 (28–55)	30 (17–41)	38 (28–52)	41 (31–53)	55 (41–78)	< 0.001
TIBC (µg/dL)	262 (225–316)	313 (253–383)	255 (239–308)	253 (219–297)	234 (196–274)	< 0.001
TSAT (%)	15.8 (9.6–22.1)	9.1 (5.9–14.7)	14.0 (9.4–20.2)	16.9 (12.6–22.3)	22.3 (17.0–43.0)	< 0.001
Ferritin (μg/L)	114 (54–244)	33 (24–81)	115 (74–200)	136 (76–242)	275 (135–442)	< 0.001
Ret-He (pg)	32.0 (29.4–34.3)	26.3 (23.3–27.6)	31.3 (30.9–31.8)	32.8 (32.6–33.7)	36.5 (35.6–38.4)	< 0.001
β-blockers, *n* (%)	91 (64)	21 (58)	25 (68)	23 (70)	22 (61)	0.168
ACE-Is/ARBs, *n* (%)	82 (58)	20 (56)	25 (68)	22 (67)	15 (42)	0.093
Ca blockers, *n* (%)	48 (34)	13 (36)	8 (22)	12 (36)	14 (39)	0.384
Loop diuretics, *n* (%)	65 (46)	14 (39)	21 (57)	21 (64)	9 (25)	0.005

Continuous variables are presented as median and interquartile range, and categorical variables are presented as number (percentage). *BMI* body mass index, *SBP* systolic blood pressure, *HR* heart rate, *Past HF* past hospitalization for heart failure, *HT* hypertension, *DM* diabetes mellitus, *AF* atrial fibrillation, *OMI* old myocardial infarction, *CVD* cerebrovascular disease, *eGFR* estimated glomerular filtration rate, *LVEF* left ventricular ejection fraction, *BNP* brain natriuretic peptide, *Alb* albumin, *Hb* hemoglobin, *MCV* mean corpuscular volume, *MCH* mean corpuscular hemoglobin, *MCHC* mean corpuscular hemoglobin concentration, *TIBC* total iron binding capacity, *TSAT* transferrin saturation, *Ret-He* reticulocyte hemoglobin equivalent, *ACE-I* angiotensin-converting enzyme inhibitor, *ARB* angiotensin II receptor blocker.

**Figure 1:**
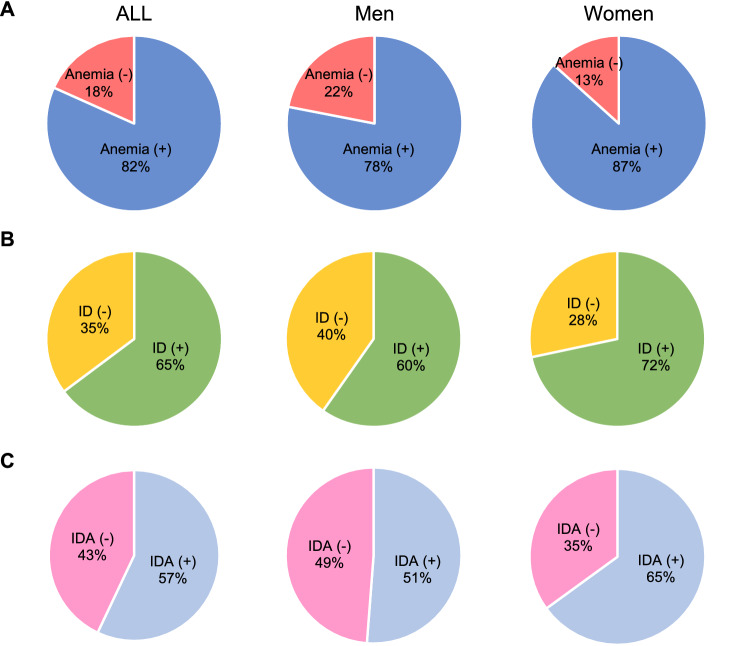
Prevalence rate of anemia, iron deficiency, and iron deficiency anemia in HF patients. Prevalence rate of (**A**) anemia, (**B**) ID, and (**C**) IDA in all HF patients. *ID* iron deficiency, *IDA* iron deficiency anemia.

Overall, using World Health Organization (WHO) criteria^12^, 82% of patients were shown anemia in this study. 78% of men and 87% of women had anemia (Fig. 1A). On the other hand, according to current guideline definition for ID, 65% patients have ID in all patients. The prevalence rate of ID was 60% in men and 72% in women (Fig. 1B). 57% of patients had IDA (men, 51% and women, 65%) (Fig. 1C).

### Relationship of Ret-He levels with parameters of iron metabolism in HF patients

**Figure 2:**
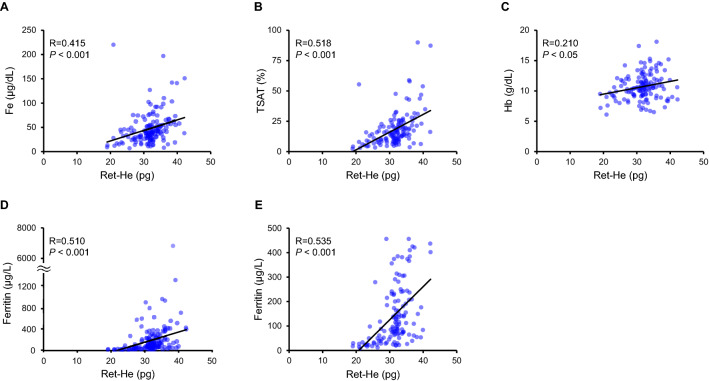
Correlation between Ret-He levels and serum iron, TSAT, serum ferritin, and Hb levels in HF patients. Correlation between Ret-He levels and (**A**) serum iron, (**B**) TSAT, (**C**), Hb, (**D**) ferritin, and (**E**) an expanded Y-axis in ferritin 0-500 μg/L in HF patients. *TSAT* transferrin saturation, *Hb* hemoglobin.

To investigate the clinical utility of Ret-He in HF patients, we next evaluated the correlation of Ret-He levels with serum iron, TSAT, ferritin, and Hb levels in HF patients. Ret-He levels were directly correlated with serum iron, TSAT, Hb, and ferritin levels in HF patients (Fig. 2A–D). In addition, to further allow interpretation of the data, we performed an additional analysis based on an expanded Y-axis in ferritin 0-500 μg/L, and a correlation between Ret-He and ferritin levels was observed (Fig. 2E).

### Comparison of Ret-He levels according to iron status in HF patients

**Figure 3:**
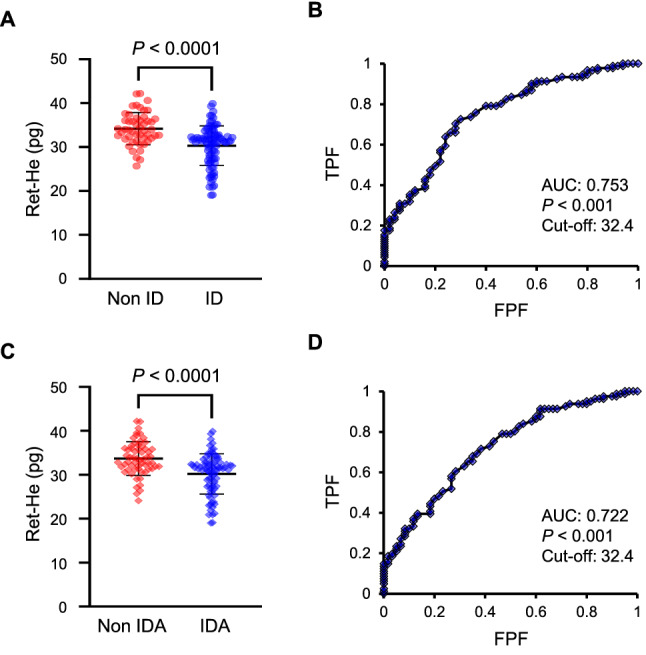
Ret-He levels in HF patients with and without iron deficiency. (**A**) Ret-He levels in HF patients with (*n* = 92) and without ID (*n* = 50). (**B**) ROC curve of Ret-He to screen for ID. (**C**) Ret-He levels in HF patients with (*n* = 82) and without (*n* = 60) IDA. (**D**) ROC curve of Ret-He to screen for IDA. *ID* iron deficiency, *IDA* iron deficiency anemia, *AUC* area under the curve, *TPF* true positive fraction, *FPF* false positive fraction.

We then evaluated Ret-He levels in HF patients according to current guideline definition for ID. The median Ret-He levels were significantly lower in HF patients with ID than in those without ID (30.3 versus 34.2 pg, *P* < 0.0001) (Fig. 3A). We next assessed the diagnostic accuracy of Ret-He to screen for ID in HF patients by receiver-operating characteristic (ROC) analysis. The area under the curve (AUC) for Ret-He to screen for ID was 0.753 and the cut-off value for Ret-He to screen for ID was 32.4 pg (true positive fraction: 0.725, 1-false positive fraction: 0.700) (Fig. 3B). In addition, we investigated Ret-He levels in HF patients according to definition for IDA. Ret-He levels were significantly lower in HF patients with IDA than in those without IDA (30.2 versus 33.7 pg, *P* < 0.0001) (Fig. 3C). And, the AUC for Ret-He to screen for IDA was 0.722. Similar to ID, the cut-off value for Ret-He to screen for IDA was 32.4 pg (true positive fraction: 0.716, 1-false positive fraction: 0.617) in HF patients (Fig. 3D).

### Prognostic associations of Ret-He levels in HF patients

**Figure 4:**
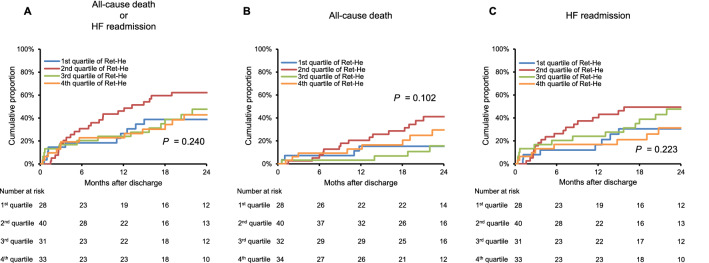
Survival curves for all-cause death or heart failure readmission according to quartile of Ret-He levels in HF patients. Kaplan–Meier analysis for (**A**) all-cause death or HF readmission, (**B**) all-cause death, and (**C**) HF readmission in HF patients by quartile of Ret-He levels.

ID is associated with an adverse prognosis in HF patients^3,5^, we thus assessed prognostic associations of Ret-He levels in HF patients. During a median follow-up of 21.7 months, composite outcomes (all-cause death or HF readmission) occurred in 61 patients. We first investigated prognosis in HF patients according to quartile of Ret-He levels; however, there was not significant different prognosis among the groups (Fig. 4A–C).

**Figure 5:**
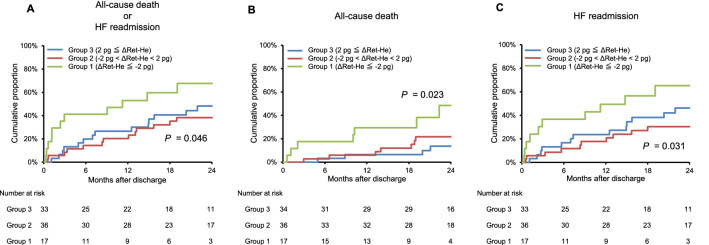
Survival curves for all-cause death or heart failure readmission according to a three-group classification of change in Ret-He levels between admission and discharge in HF patients. Kaplan–Meier analysis for (**A**) all-cause death or HF readmission, (**B**) all-cause death, and (**C**) HF readmission in HF patients by a three-group classification of change in Ret-He levels between admission and discharge (ΔRet-He).

Then, we investigated prognostic associations of Ret-He levels in HF patients, whose Ret-He levels were measured both at admission and discharge, by change in Ret-He levels between admission and discharge (ΔRet-He). The distribution of ΔRet-He is shown in Supplemental Fig. 1. As there were patients with little change in Ret-He levels between admission and discharge, we performed a prognostic analysis based on a three-group classification of change in Ret-He levels (group 1: ΔRet-He ≦ − 2 pg, group 2: − 2 pg < ΔRet-He < 2 pg, and group 3: 2 pg ≦ ΔRet-He). Of note, a statistically significant worse prognosis was observed in group 1 than in the other groups (Fig. 5A–C). The patient characteristics by a three-group classification of change in Ret-He levels are shown in Table 2. The median body mass index, TSAT, Ret-He, the prevalence of atrial fibrillation, and the use of Ca blockers were different among the groups.

**Table 2:** Patient characteristics on admission according to a three-group classification of change in Ret-He levels between admission and discharge.

Variable	Group 1 ΔRet-He ≦ − 2 pg (*n* = 17)	Group 2 − 2 pg < ΔRet-He < 2 pg (*n* = 36)	Group 3 2 pg ≦ ΔRet-He (*n* = 35)	*P* value
Age (years)	78 (74–82)	76 (72–84)	75 (68–82)	0.303
Men, *n* (%)	10 (59)	23 (64)	25 (71)	0.631
BMI (kg/m^2^)	21.4 (19.6–23.9)	22.6 (21.0–24.9)	24.0 (22.1–27.9)	0.044
SBP (mmHg)	131 (126–143)	119 (109–142)	129 (124–148)	0.147
HR (bpm)	69 (61–81)	81 (67–98)	85 (69–105)	0.081
NYHA functional class III/IV, *n*	16 (94)	33 (92)	34 (97)	0.608
Past HF, *n* (%)	8 (47)	15 (42)	20 (57)	0.421
HT, *n* (%)	16 (94)	30 (83)	24 (69)	0.077
DM, *n* (%)	6 (35)	19 (53)	14 (40)	0.393
AF, *n* (%)	4 (24)	8 (22)	17 (49)	0.040
OMI, *n* (%)	8 (47)	11 (31)	10 (29)	0.381
CVD, *n* (%)	2 (12)	3 (8)	5 (14)	0.731
eGFR (mL/min/1.73m^2^)	31 (19–50)	51 (32–69)	57 (38–67)	0.077
LVEF (%)	36 (27–55)	37 (29–60)	35 (24–51)	0.504
BNP (pg/mL)	1050 (352–2470)	644 (426–974)	640 (519–1390)	0.325
Alb (g/dL)	3.2 (2.9–3.9)	3.4 (3.1–3.7)	3.3 (3.1–3.7)	0.563
Hb (g/dL)	10.3 (8.7–10.9)	10.7 (10.1–11.4)	10.6 (9.5–12.0)	0.403
MCV (fL)	93.7 (88.9–98.6)	92.6 (88.4–97.1)	92.1 (86.4–96.0)	0.450
MCH (pg)	31.1 (30.0–33.3)	30.6 (28.7–32.2)	30.6 (27.7–31.4)	0.315
MCHC (%)	33.3 (32.7–33.6)	32.8 (32.2–33.7)	33.0 (31.5–33.7)	0.271
Fe (µg/dL)	54 (38–65)	44 (35–62)	34 (24–53)	0.132
TIBC (µg/dL)	235 (201–286)	255 (232–332)	297 (233–344)	0.073
TSAT (%)	19.2 (13.0–34.9)	18.2 (12.5–22.8)	11.1 (8.3–19.3)	0.034
Ferritin (μg/L)	176 (89–437)	121 (63–228)	79 (31–193)	0.052
Ret-He (pg)	36.6 (32.9–39.0)	32.3 (31.1–34.2)	31.2 (26.6–32.3)	< 0.001
β-blockers, *n* (%)	13 (76)	23 (64)	23 (66)	0.646
ACE-Is/ARBs, *n* (%)	10 (59)	22 (61)	21 (60)	0.987
Ca blockers, *n* (%)	10 (59)	11 (31)	8 (23)	0.032
Loop diuretics, *n* (%)	5 (29)	20 (56)	18 (51)	0.191

Continuous variables are presented as median and interquartile range, and categorical variables are presented as number (percentage). *ΔRet-He* the change of Ret-He levels between admission and discharge, *BMI* body mass index, *SBP* systolic blood pressure, *HR* heart rate, *Past HF* past hospitalization for heart failure, *HT* hypertension, *DM* diabetes mellitus, *AF* atrial fibrillation, *OMI* old myocardial infarction, *CVD* cerebrovascular disease, *eGFR* estimated glomerular filtration rate, *LVEF* left ventricular ejection fraction, *BNP* brain natriuretic peptide, *Alb* albumin, *Hb* hemoglobin, *MCV* mean corpuscular volume, *MCH* mean corpuscular hemoglobin, *MCHC* mean corpuscular hemoglobin concentration, *TIBC* total iron binding capacity, *TSAT* transferrin saturation, *Ret-He* reticulocyte hemoglobin equivalent, *ACE-I* angiotensin-converting enzyme inhibitor, *ARB* angiotensin II receptor blocker.

To investigate the prognostic impact of ΔRet-He, we finally performed univariate and multivariate Cox regression analyses for prognosis in these patients. In the univariate model, ΔRet-He ≦ − 2 pg was associated with a worse outcome, while this tended to be associated with a worse outcome in a multivariate analysis (Table 3).

**Table 3:** Univariate and multivariate Cox regression analysis for composite outcomes.

	Univariate model		Multivariate model*	
	HR (95% CI)	*P* value	HR (95% CI)	*P* value
ΔRet-He **≦ − **2 pg	2.32 (1.15–4.68)	0.019	2.19 (0.88–5.49)	0.093
** − **2 pg < ΔRet-He < 2 pg	0.60 (0.31–1.18)	0.139	0.61 (0.28–1.29)	0.195
2 pg **≦** ΔRet-He	0.93 (0.48–1.79)	0.817	1.04 (0.47–2.30)	0.930

*Model adjusted for age, male, body mass index at discharge, brain natriuretic peptide levels at discharge, the prevalence of atrial fibrillation, and the use of Ca blockers. *ΔRet-He* the change of Ret-He levels between admission and discharge, *HR* hazard ratio, *CI* confidence interval.

## Discussion

This study showed for the first time that Ret-He has the clinical utility as a simple marker for ID in HF patients. Ret-He levels were directly correlated with serum iron and ferritin levels and with TSAT. Moreover, Kaplan–Meier analysis demonstrated that increases or decreases in Ret-He between admission and discharge were associated with a worse outcome in HF patients.

The gold-standard for measurement of ID is bone marrow histology. Although there are many definitions of ID based on circulating biomarkers, it is unknown which marker is best. Serum ferritin levels are commonly used to evaluate iron status; however, as ferritin is an acute phase protein, serum ferritin levels increase in the presence of chronic inflammation. As chronic inflammation is associated with the pathogenesis of HF, it should be taken into consideration. In fact, previous studies have shown that higher serum ferritin levels are associated with an adverse prognosis in HF patients^13,17^. Thus, cut-off values of these markers for ID should be reconsidered for HF patients. In this regard, Ret-He can be easily obtained, it thus may be advantageous for the screening for ID in patients with HF.

Ret-He can reveal the current iron status that are clinically significant. Of note, Ret-He levels were directly correlated with standard parameters of iron metabolism in HF patients. In this study, there were a few patients whose TSAT was about 90%. These patients had liver congestion or poor nutrition due to severe HF, which resulted in low transferrin production and high TSAT. In addition, the cut-off value for Ret-He to screen for both ID and IDA as defined in current international guidelines was 32.4 pg. These results indicate that Ret-He could reflect iron status and lead more easily applicable definition to screen for ID and IDA in HF patients. Ret-He can be easily measured in the same tube of cellular blood analysis, and the costs of Ret-He measurement are also economical. Taken together, these results suggest that Ret-He has the clinical utility as a simple marker for ID in HF patients. As the reports of improved clinical symptom with intravenous (IV) iron administration focus on anemia and ID in HF patients^14–16^, the measurements of Ret-He levels is considered to have an important perspective in HF patients.

A recent study has shown that there was no association between current guideline definition for ID and mortality in HF patients^17^. Similarly, in this study, ID defined by current guideline was not associated with prognosis in HF patients (*P* = 0.472 for all-cause death or HF readmission, *P* = 0.926 for all-cause death, *P* = 0.282 for HF readmission). Then, we evaluated the prognostic impact of Ret-He levels in HF patients. However, there was not a significant difference on prognosis according to quartile of Ret-He levels. We then investigated the prognostic impact of Ret-He in HF patients, by change in Ret-He levels between admission and discharge. Of note, by a prognostic analysis based on a three-group classification of change in Ret-He levels, a worse outcome was observed in patients with ΔRet-He ≦ − 2 pg than in the other groups. Also, multivariate analysis showed that ΔRet-He ≦ − 2 pg tended to be associated with a worse outcome. These results indicate that ΔRet-He ≦ − 2 pg even after optimal HF treatment is associated with a worse outcome in HF patients. To the best of our knowledge, this study showed for the first time that the change in Ret-He levels between admission and discharge was associated with prognosis in HF patients. Treatment of ID by monitoring Ret-He levels may lead to improve prognosis in HF patients. Recent clinical studies have shown that IV iron administration is beneficial for clinical symptom in HF patients with reduced ejection fraction with ID^14–16^. However, the effects of IV iron administration on prognosis in HF patients has been entirely unknown. Iron administration may provide a benefit for prognosis particularly in HF patients with ΔRet-He ≦ − 2 pg. In conclusion, these findings support the clinical utility of Ret-He to assess ID in HF patients. However, the clinical utility of Ret-He for identifying ID and predicting response to IV iron administration and prognosis for patients with HF requires further investigation.

### Study limitations

This study is a small single-centered and relatively small number study. Further validation in the future is needed.

## Methods

### Study protocol

We studied 207 consecutive patients who were hospitalized for acute decompensated HF according to the Framingham criteria^18^ between December 1, 2017 and October 31, 2018 at Hyogo Medical University Hospital. Patients with renal failure with estimated glomerular filtration rate < 10 mL/min/1.73 m^2^ or receiving hemodialysis (*n* = 18), leukemia (*n* = 1), autoimmune hemolytic anemia (*n* = 2), or active malignancy (*n* = 12) were excluded. In addition, we excluded 25 participants whose missing all the required iron indices and 7 participants who were lost to follow-up, which left a final analytical cohort of 142 participants (Supplementary Fig. 2). The patients’ demographic data including co-morbid conditions, clinical signs, laboratory and echocardiographic data, in-hospital treatment including oral and intravenous medication, and length of hospital stay were obtained. Echocardiography was performed by sonographer and LVEF was defined with the modified Simpson method. Hypertension was defined as systolic blood pressure ≧ 140 mmHg or diastolic blood pressure ≧ 90 mmHg or receiving anti-hypertensive drugs. Diabetes mellitus was defined as glycated hemoglobin ≧ 6.5%, casual blood glucose ≧ 200 mg/dL, or fasting blood glucose ≧ 126 mg/dL or receiving oral hypoglycemic agents and/or insulin. The presence of old myocardial infarction and cerebrovascular disease was defined based on history, clinical presentation, examinations, and medications.

Anemia was defined according to WHO criteria (Hb levels < 13 g/dL in men, Hb levels < 12 g/dL in women). ID was defined as serum ferritin levels < 100 μg/L, or serum ferritin levels 100–299 μg/L with TSAT (serum iron levels / TIBC × 100) < 20%^7,14,19^. IDA was defined by serum ferritin < 100 μg/L, or between 100 and 299 μg/L with TSAT < 20% and Hb levels below WHO criteria of anemia. Ret-He levels were determined by an automated hematology analyzer (XN-9100^®^ or 1000^®^, Sysmex, Kobe, Japan). Blood samples were collected within 24 h from the admission. Laboratory parameters were measured at admission and discharge.

The primary outcome was defined as a composite endpoint of all-cause death or HF readmission and prospectively recorded. Follow-up was performed at discharge, and after discharge by direct contact with patients, telephone interview of patients or, if deceased, of family members, and mail, by investigators. Informed consent were written from all patients. These procedures for informed consent and enrolment were in accordance with the detailed regulations regarding informed consent described in the guidelines, and this study has been approved by the ethics committee of Hyogo Medical University (authorization numbers 3549).

### Statistical analysis

Continuous variables were presented as median and interquartile range. Continuous variables were compared using 1-way analysis of variance or Kruskal–Wallis test. Continuous variables were tested for normality using the Shapiro–Wilk test. Categorical variables were made by chi-squared test or Fisher’s exact test for dichotomous variables, when appropriate. The correlations of Ret-He levels with serum iron, TSAT, ferritin, and Hb levels were assessed by Spearman correlation analysis*.* The cumulative incidence of a composite outcome (all-cause death or HF readmission) was estimated using Kaplan–Meier analysis. ROC analysis was performed to assess the potential capabilities of Ret-He as a marker for ID and IDA, computing the ROC curve and its AUC. The results of this analysis can be shown as the ROC curve, in which the sensitivity is plotted against the value of 1-specificity. To investigate the prognostic impact of change in Ret-He levels, covariates which were closely related to change in Ret-He levels (age, sex, body mass index, BNP, the prevalence of atrial fibrillation, and the use of Ca blockers) were chosen in this study. All tests were two tailed, and a value of *P* < 0.05 was considered statistically significant. All analyses were performed with R version 3.3.2. All methods were performed in accordance with relevant guidelines and regulations.

## Supplementary Information

Supplementary Information 1.

Supplementary Information 2.

## Data Availability

The data that support the findings of this study are available from the corresponding author upon reasonable request.
