# Supplementary material for: Clinical utility of reticulocyte hemoglobin equivalent in patients with heart failure
Source: Sci Rep. 2022 Aug 17;12:13978. doi: 10.1038/s41598-022-18192-x (PMC9385695; doi:10.1038/s41598-022-18192-x)
Supplement: Supplementary file 1 — Supplementary Information 1. [file 41598_2022_18192_MOESM1_ESM.pdf]

Supplemental Figure 1

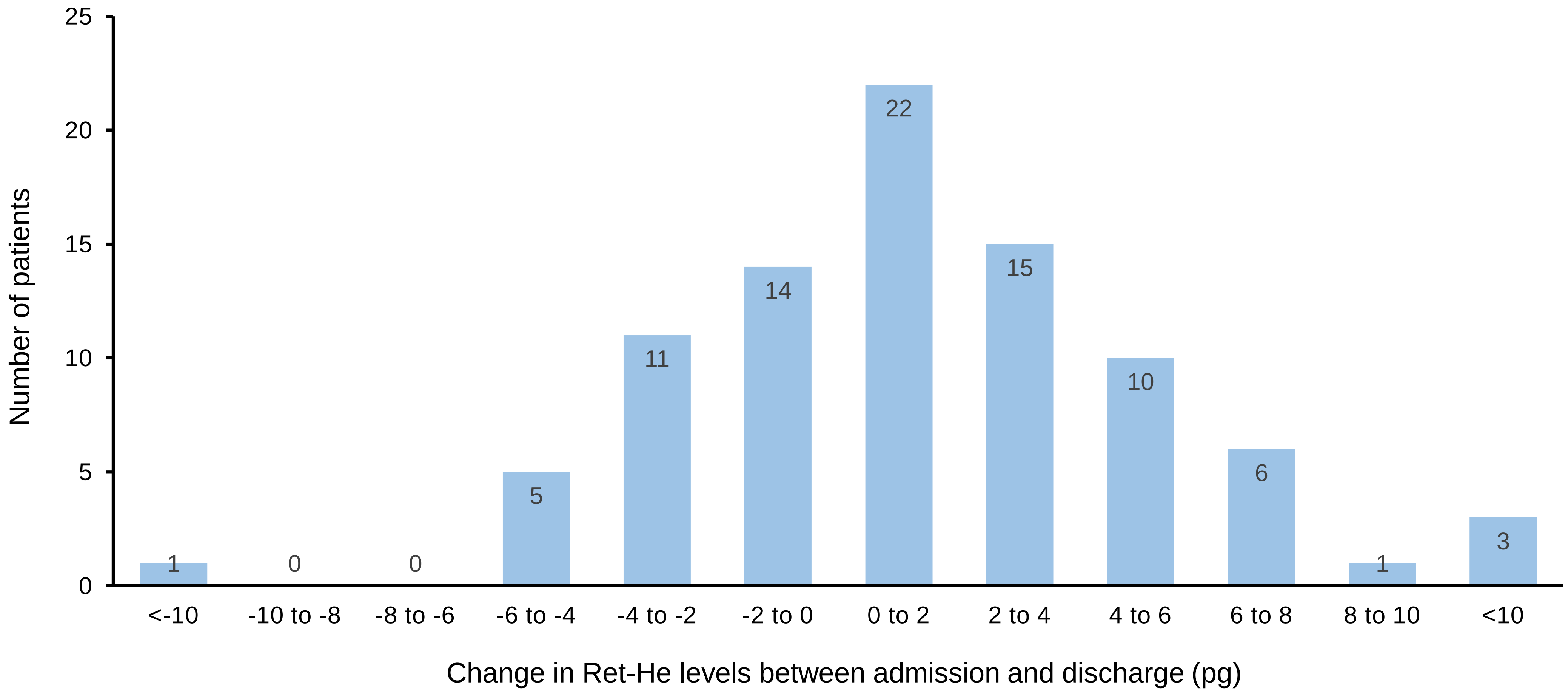

The distribution of change in Ret-He levels between admission and discharge in HF patients.
