# Supplementary figures and images for: Clinical utility of reticulocyte hemoglobin equivalent in patients with heart failure
Source: Sci Rep. 2022 Aug 17;12:13978. doi: 10.1038/s41598-022-18192-x (PMC9385695; doi:10.1038/s41598-022-18192-x)

**Supplemental Figure 2**

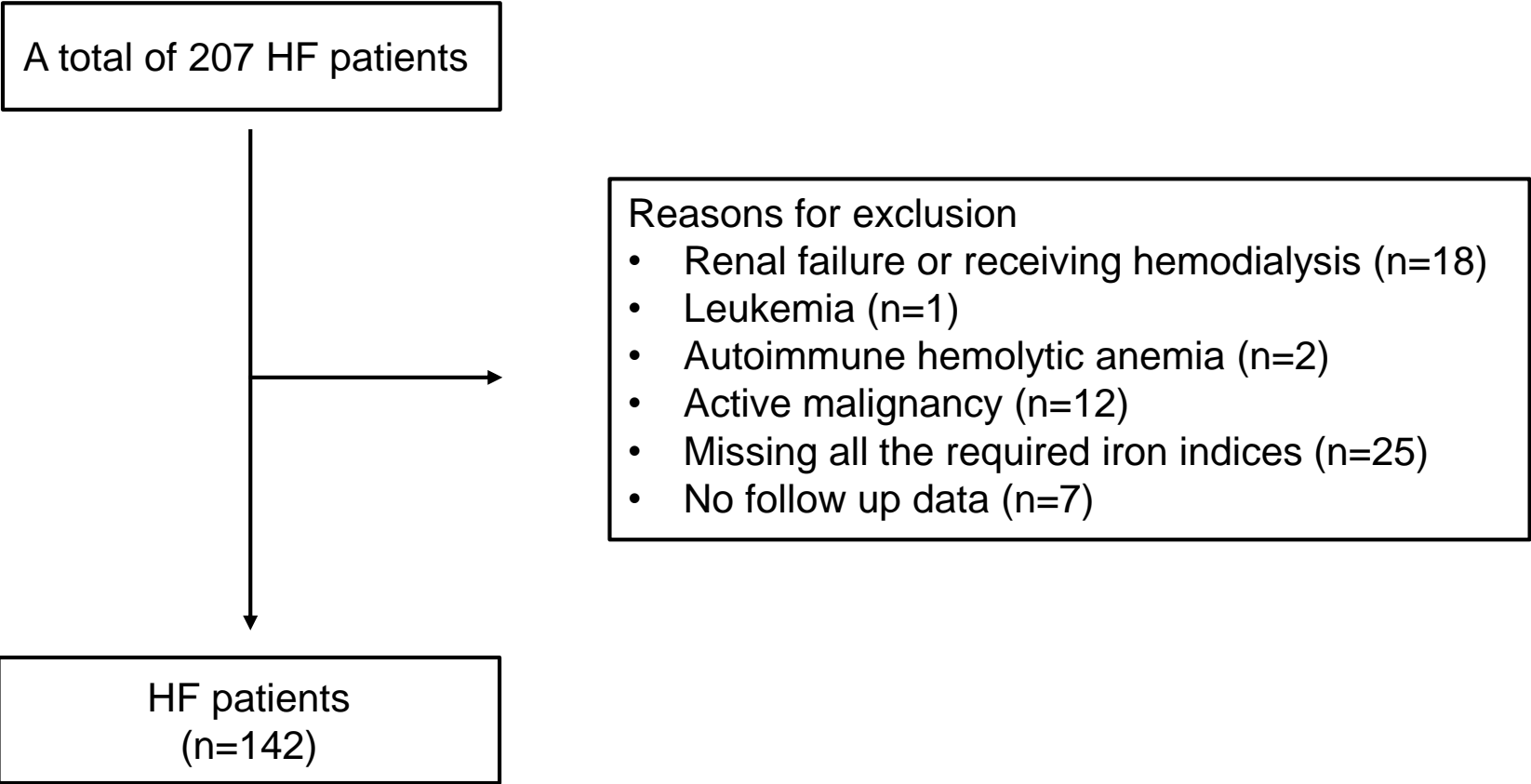

**Study flow chart.**

Supplement: Supplementary file 2 — Supplementary Information 2. [file 41598_2022_18192_MOESM2_ESM.pdf]
